# Practical approaches for engaging the public in a population-level data analysis project.

**DOI:** 10.23889/ijpds.v9i5.1671

**Published:** 2024-09-18

**Authors:** Sabella Yussuf-Homenauth, Laura Ferreira-Legere, Elise Leong-Sit, Astrid Guttmann, Michael J. Schull, Michael Campitelli, J Michael Paterson, The ICES Public Advisory Council

**Affiliations:** 1 ICES; 2 Hospital for Sick Children; 3 Dalla Lana School of Public Health; 4 University of Toronto; 5 Sunnybrook Health Sciences Centre; 6 McMaster University

## Objective and Approach

Stewards of population-level data have an obligation to serve the public interest. As one such publicly funded data steward, our Public Advisory Council (PAC) co-led and determined the focus of an analysis project using population-level administrative health data. We describe our engagement strategies herein.

## Approach

The 20-member PAC was involved in each stage of the project, from research question formulation to knowledge translation planning over 18 months, consisting of 12 meetings with both large and smaller groups. Our approach to engagement applied the International Association for Public Participation’s Framework as the foundation for an ‘empowerment’ level of engagement and adapted approaches from the James Lind Alliance and the ‘Plan-Do-Study-Act’ cycle.

## Results

The PAC chose to focus their analysis project on factors related to mental health and addiction service use. Four strategies were used and co-designed by members to foster engagement throughout: providing education and guidance, shared and guided brainstorming, building consensus, and responsiveness to feedback and evaluation. PAC members directed how and when these strategies were used, with challenges and lessons learned currently being co-developed into a publicly accessible report.

## Conclusion

Our work demonstrates the importance and value of public-driven research outputs and the feasibility of integrating public members in the work of data stewards.

## Implications

The insights and practical strategies generated from this project will be used to guide effective engagement of the public for future analysis projects and to improve trust and social license for other initiatives using population-level data.

